# Retraction: Rabi, A.; Khader, Y.; Alkafajei, A.; Aqoulah, A.A. Sanitary Conditions of Public Swimming Pools in Amman, Jordan. *Int. J. Environ. Res. Public Health* 2008, *5*, 152–157

**DOI:** 10.3390/ijerph13070646

**Published:** 2016-06-28

**Authors:** International Journal of Environmental Research and Public Health Editorial Office

**Affiliations:** Klybeckstrasse 64, 4057 Basel, Switzerland; ijerph@mdpi.com

The title article [1] is a duplicate publication of [2]. This was due to an error made by the editorial office. We very much regret that this was not detected and no action has been taken until now, and apologize to the authors and readers of the *International Journal of Environmental Research and Public Health* for any inconvenience. The article [1] will be marked as retracted.
